# Legal Notes for Institutional Workers

**Published:** 1911-11-11

**Authors:** 


### LEGAL NOTES FOR INSTITUTIONAL WORKERS.

(The Editor will be pleased to answer Legal queries in this column.)

### SICK RELIEF OUTSIDE MUNICIPAL AREAS.

The rapidly extending responsibilities of local authorities often make it necessary for these bodies to consider how far they are entitled to establish or support hospitals outside their districts for the relief of the sick. In a recent number of the *Justice of the Peace* the following question is propounded: "Is it legal for a local authority, say an urban or a rural district council, to annually contribute a subscription to an ordinary hospital for women outside the district of the authority?" The answer is to be found in s. 131 of the Public Health Act, 1875, which provides that a local authority may contribute to a hospital outside its district an annual sum in consideration of the hospital receiving the sick inhabitants of its district. There appears to be no reason why provision should not be made for women alone. At the same time, a local authority would have no power to provide an infirmary—*i.e.* a place for aged and decrepit persons.

### HOSPITALS AND WORKMEN'S COMPENSATION.

A case of unusual importance to hospital authorities was recently decided at Manchester County Court. While the Workmen's Compensation Act is specially applied to certain industrial diseases, it is still open to a judge to find that any disease is the result of an accident. It appears that the mortuary porter at the Monsall Fever Hospital contracted scarlet fever. He had recently had influenza, and the fever was followed by nephritis, which totally incapacitated him from work. Upon his making a claim for compensation a doctor called on his behalf said that if the body of a person who had died from scarlet fever were taken into the mortuary, as several had been, it would be a hotbed of the disease. In these circumstances the Judge held that the claimant had been the victim of an accident, and awarded him compensation at the rate of 15s. a week during incapacity. If this decision stands—and we notice that a stay of execution was granted pending an appeal—it will seriously increase the financial responsibility of those who are in charge of fever hospitals. The nurses and the ward-maids are continually exposed to infection; and the question whether infection was caused "in the course of the employment" is merely one of fact for the County Court Judge to decide.

### SCARLET FEVER AND THE LAW OF NEGLIGENCE.

When does a patient who has had scarlet fever cease to be infectious? This question was discussed in a recent case at the Hanley County Court. An action was brought against Dr. Phillips, of the Bucknall Isolation Hospital, for having negligently allowed a boy to leave too soon. The plaintiff, who was the boy's step-father, became infected with scarlet fever three days later. According to the evidence, the boy had no running from the nose when he left, but he seems to have caught cold the following day. The plaintiff's wife admitted that she received printed instructions warning her against allowing the boy to catch cold. According to Dr. Phillips' evidence there was only a little roughness on the boy's heel when he was discharged. He did not regard the later stages of desquamation as infectious, although, as a matter of fact, when there was much peeling he did not discharge patients, because of the feeling which existed as to its infectivity. Dr. Frank Shufflebotham, who was called on the plaintiff's behalf, quoted a passage from an article by Dr. Caiger in *Allbutt's System of Medicine*, in which it is stated that "desquamation . . . may persist and be contagious for a period of three or four months." He admitted, however, that this view is modified in a later edition. The Medical Officer of Health for Staffordshire having stated that the present views of the public health world were that the peeling of the epidermis was not a source of infection, Judge Ruegg said he was bound to give judgment for the defendant, but added that the evidence with regard to desquamation was new to him. Dr. Phillips, who was defended by the London and Counties Medical Protection Society, is to be congratulated on the result. If patients had to be kept in hospital for the period referred to by Dr. Caiger it is difficult to see how sufficient accommodation could be provided. The parents and friends of discharged patients must be expected to take the precautions against cold which are so necessary in the case of a patient who has suffered from scarlet-fever.

